# Supplementary material for: Comparing dual oral agents plus insulin vs. Triple oral agents in uncontrolled type II diabetes: A pilot study
Source: PLoS One. 2024 Nov 21;19(11):e0311435. doi: 10.1371/journal.pone.0311435 (PMC11581212; doi:10.1371/journal.pone.0311435)
Supplement: Attachment — Submitted filename: Rebuttal for comments.docx [file pone.0311435.s001.docx]

**Dear Editor and Reviewers,**

Thank you for taking the time to provide such detailed and helpful comments on our manuscript. We appreciate your constructive criticisms and suggestions, and we believe they will significantly improve the quality of our manuscript. We have revised the manuscript based on your feedback and would like to provide the following responses to your concerns.

**Reviewer #1:**

The authors address a subject of crucial importance as Diabetes mellitus is increasing its prevalence each year. Despite the variety of drugs and insulin, it's still a challenge to decide the best regimen for better glycemic control and reduce its complications. Although a few studies suggest that starting with combined OHA may achieve better results, others suggest that the better approach is a shared decision with the patient; there isn't a definitive study to show whether adding a third OHA or insulin to achieve satisfactory glycemic control is better. Although a pre-post study design is less expensive than the RCT, does not require randomization, and can provide some information about the effect of the intervention alongside its use, it has some limitations as it cannot avoid bias either state for sure if the outcome can be attributed to the procedure. Some other points need to be clarified, mainly related to the methods.

#1 In the abstract, some methods are described in the results.

Response: Thank you for the correction, suggested changes are done in the manuscript.

#2 Page 4, line 105: The item inclusion/exclusion criteria don't say that only patients using sitagliptin+ metformin would be included or whether there is a protocol that patients use these drugs as the first line of treatment. It would be helpful to determine whether the patients were in total doses of the medications when they started the third OHD or insulin. And explain how they began and titrated the drug or insulin dose.

Response: Thank you for the comment, the inclusion criteria comprised of patients who were on full doses of sitagliptin+ metformin as a first line therapy and their diabetes was uncontrolled based on HbA1c values. The suggested changes have been done.

#3Page 5-6, Table 1: I would not expect that Table 1 shows the baseline treatment; all patients in group 1 were already on a 3-drug regimen. Was that a mistake?

Response: Thank you for the highlight this issue. This was a mistake which was overlooked at time of submission. The correction has been made in the manuscript and the details are removed from Table 1 to avoid confusion to readers. The details of group 1 and group 2 are given in text after table 1.

#4 In the results, it would be helpful to include, if available, the data on dropouts or compliance with the treatment.

Response: Thank you for comment, the compliance was ensured by principal author as the medication were given to patients free of cost and on monthly basis the patients were instructed to meet the principal author to get the next month free doses of their medication. This also help to avoid the drop out of the patients in the study.

#5 From page 10 to the end, the lines are not numbered

Response: Thank you for comment, the line numbers are assigned to the whole manuscript as suggested.

#6 Page 11, in the discussion, what do you mean by...' despite aggressive regimens?

Response: Thank you for comment and highlighting this word, to avoid confusion to readers this has been changes to “triple OHAs regimen”.

#7 On page 11, the authors attribute the weight loss to metformin. Is there any other data to support that? All patients were using metformin at the baseline, and 20% of group 1 started taking Empagliflozin.

Response: Thank you for comment, the statement has been modified and updated to “Similarly, in Group 1 the weight reduction may be attributed to empagliflozin as 20% of the patients were using empagliflozin as a third OHAs, which are consistent with findings of another study [51]”.

#8 The manuscript needs corrections in the grammar and punctuation.

Response: Thank you for comment, the whole manuscript has been thoroughly edited by native English speaker.

**Reviewer #2:**

1. On the scope and title: The title is too long and needs adequate shortening. It should be guided by the scope of the study. Commonly we write as “type II Diabetes Mellitus” not “type 2 Diabetes Mellitus”.

Response: Thank you for comment, the title is shorten as suggested to “**Comparing Dual Oral Agents Plus Insulin vs. Triple Oral Agents in Uncontrolled Type II Diabetes: A Pilot Study”** and the “type 2 Diabetes Mellitus” is replaced by “type II Diabetes Mellitus” in the manuscript. We included “pilot study” in title as it is suggested by one of the reviewers.

2. On the abstract: The abstract lacks clarity and logical presentation E.g., the type of study design and analysis is missed. The result and the conclusion are inconsistent.

Response: Thank you for the correction, suggested changes are done in the manuscript. The results and conclusion were rewritten again as suggested.

3. On the background: It is little bit far from the study objective. E.g., Very few points are described regarding the treatment of uncontrolled type II Diabetes and its outcome. In addition, it contains mixed of ideas and lacks linking between points. E.g., It directly jumps from global prevalence to Pakistan. Most importantly, the section lacks the main problem statement of the study.

Response: Thank you for comment, the background section has been updated as suggested.

4. On the methods section: Similarly, the methods section is incomplete for the study design, process of measurements, the sampling procedure and selection of participants as well as the type of analysis. Ethics should be presented in detail in line with the protocol of the study.

Response: Thank you for comment, the method section has been updated as suggested. However, the ethics approval details are not mentioned as the journal requested us to remove these details at the time of peer-review process.

5. On the result: it lacks logical flow and brief presentation including lack of self-explanatory of tables.

Response: Thank you for the comment, the result section is rewritten again to avoid confusion to the readers.

6. The discussion is inadequate and needs rich explanation.

Response: Thank you for the comment, the discussion section is updated as suggested.

7. Others: In addition, lacks consistency E.g., you have used inclusion criteria in line 105-109 and ‘illegibility criteria’ in line 115.

Response: Thank you for the highlight, the correction is done in manuscript as suggested.

**Reviewer #3:**

Thank you, Editor, for inviting me to review this research paper. Authors, your research paper provides valuable insights in the field. However, some aspects need clarification and additional details to enhance the comprehensibility and robustness of your work. Below are my comments and suggestions:

Abstract:
1. Aim: Please rewrite the aim of the study to be clearer.

Response: Thank you for comment, the aim is rewritten again as suggested.

2. Glycemic Episodes: In the results section of the abstract, add the p-value (p=0.004) at the end of the sentence discussing glycemic episodes for better clarity.

Response: Thank you for comment, the suggested changes has been done.

Introduction:
3. Line 71: It would be interesting to include the prevalence of glycemic episodes among men and women in Pakistan to provide context and relevance to your study.

Response: Thank you for comment, the suggested changes has been done.

Methods:
4. Rationale for Criteria: Explain why you chose those with HbA1c more than 7 as the threshold. What is the rationale behind this choice? Educating the readers on this decision would enhance understanding.

Response: Thank you for comment, the laboratory standard use in this study showed a HbA1c range: Normal: 4-6.5, Pre-diabetes: 6.6-7, diabetes: >7.1-8.0. we used this criterion and this was the rationale behind this choice.

5. Participant Assignment: Clarify how participants were assigned to group 1 and group 2. Were they randomly allocated? This information is crucial for assessing the validity of your study design.

Response: Thank you for comment, the statement is updated in the manuscript as below: “After meeting the inclusion criteria, the patients were randomly assigned into two groups”.

6. Study Awareness: Describe how patients were informed about the study. This could include recruitment strategies.

Response: Thank you for comment, the statement is updated in the manuscript as below: “The patients were approached and the purpose of the study was explained to them in Prescence of consultant endocrinologist. Those who were willing to participate an informed written consent was obtained from them”.

7. Literature Support: Include literature that supports the notion that three months is a sufficient period to observe changes in patients' HBA1c levels.

Response: Thank you for comment, the suggested changes has been done. “HbA1c is a marker of the average glucose levels spread over a two- to three-month period [25]”.

8. Data Distribution: Indicate whether your data were normally distributed. This affects the choice of statistical tests and the interpretation of results.

Response: Thank you for comment, the normality of the data was checked by using Shapiro-Wilk Test and the data showed normal distribution. Keeping in mind the normality of data, the data was presented as mean and SD. Parametric test were performed in this study as data was normally distributed.

Results:
9. Terminology: On line 157, change "the majority" were females to reflect the actual percentage. Referring to 52% as a majority is misleading. Similarly, clarify that 50% does not constitute the majority.

Response: Thank you for comment, the result section is rewritten again to avoid confusion to readers.

10. Follow-Up: Specify if there were any losses to follow-up. If yes, provide the number of patients lost and discuss the potential impact on your results.

Response: Thank you for comment, there was no drop out in this study. The principal author was responsible for giving medication free of cost to the patients and on monthly basis the patients were instructed to meet the principal author to get the next month free doses of their medication. This also help to avoid the drop out of the patients in the study.

11. Compliance: Discuss how you ensured patients' compliance with the treatment regimen. How did you confirm that they took their medications on time? I am aware that the authors mentioned this as a limitation, but did the researchers not address patient compliance at all?

Response: Thank you for comment, the principal author was responsible for giving medication free of cost to the patients and on monthly basis the patients were instructed to meet the principal author to get the next month free doses of their medication. This also helped in compliance of the patients toward their medication. However, no specific scale/instrument was utilized for evaluation of the adherence to therapy that’s why this was included as a limitation in limitation section.

12. Group Allocation: Reiterate how patients were allocated to each group. This should be clear and transparent to readers.

Response: Thank you for comment, the statement is incorporated in the manuscript as: “After meeting the inclusion criteria, the patients were randomly assigned into two groups”

13. Rationale for Grouping: Explain the rationale behind your age groups, weight, and BMI categories. Providing a justification for these groupings would help in understanding your results.

Response: Thank you for comment, the age and weight were categorized based on the distribution of patient keeping in mind a comparable number of patients in each category. However, for the BMI, WHO classification was used.

14. Table 1: The oral glycemic agents in Table 1 are confusing. Please present this information more clearly.

Response: Thank you for comment, the details are removed from table 1 and explained at end of table 1 as below:

“All the included patients in this study were on dual OHAs i.e. Sitagliptin + Metformin. The patients were assigned into two groups. In group 1, the patients were started with additional third OHA i.e. Empagliflozin or Gliclazide or Glimepiride by their consultant endocrinologist. While for the group 2 patients were started with insulin in addition to their dual OHAs i.e. Sitagliptin + Metformin”.

Discussion:
15. HbA1c Values: Refer to HbA1c as a value and not a percentage.

Response: Thank you for comment, the HbA1c are generally presented as percentages rather than value. That is why they are presented in percentages.

16. Study Limitations: Given the study limitations, particularly the lack of tracking patient compliance, consider adding "A Pilot Study" to your title to accurately reflect the scope and preliminary nature of your research.

Response: Thank you for comment, the suggested changes has been done.

Again, thank you for your time and valuable feedback. We believe that the revisions have significantly improved the manuscript and hope that it is now suitable for publication.

Regard’s

**Corresponding author**
