# Supplementary material for: Comparing dual oral agents plus insulin vs. Triple oral agents in uncontrolled type II diabetes: A pilot study
Source: PLoS One. 2024 Nov 21;19(11):e0311435. doi: 10.1371/journal.pone.0311435 (PMC11581212; doi:10.1371/journal.pone.0311435)
Supplement: Attachment — Submitted filename: Rebuttal to reviewers comments.docx [file pone.0311435.s002.docx]

**Dear Editor and Reviewers,**

Thank you for taking the time to provide such detailed and helpful comments on our manuscript. We appreciate your constructive criticisms and suggestions, and we believe they will significantly improve the quality of our manuscript. We have revised the manuscript based on your feedback and would like to provide the following responses to your concerns.

**Reply to comments**

**Reviewer #4**

This study has some limitations, which I think authors have already acknowledged in the discussion section. The authors have also addressed the majority of the other reviewers' comments. However, I have the following additional comments/suggestions to authors:

**Comment:** 1. For ease of understanding, authors should consider including a flow chart that shows the number of participants evaluated for potential enrolment into the trial and the number excluded because they did not meet the inclusion criteria or declined to participate.

**Response:** Thank you for your comment, the flowchart is added to the manuscript as Figure 1.

**Comment:** 2. Authors should also include information on the time of the day when treatment was given to participants, as well as whether the drugs were given before or after a meal. This information can be added to the methods/results section.

**Response:** Thank you for your comment. Patients were advised to take the Sitagliptin + Metformin combination tablet twice a day, either with a meal or right after eating. Empagliflozin should be taken once in the morning, along with breakfast. Glimepiride is also to be taken once daily, with breakfast. Similarly, Gliclazide is taken once daily in the morning, with a meal.

**Comment:** 3. Figure legends should be expanded to include brief descriptions of the plots depicted.

**Response:** Thank you for your comment, the suggested changes have been incorporated in the manuscript.

**Reviewer #5**

Thank you for offering me an invitation to be the reviewer of this manuscript “Comparing Dual Oral Agents Plus Insulin vs. Triple Oral Agents in Uncontrolled Type II Diabetes: A Pilot Study”. The authors had submitted a manuscript comparing dual OHA’s with Insulin and triple OHA’s in uncontrolled Type II diabetes. The selected topic becomes increasingly significant given the burden of diabetes in Asian countries.

I am giving my observations and comments for the authors to consider:
**Comment:** I observe the corresponding author in the first page is different from the one mentioned in page 1 line 18-24.

**Response:** Thank you for your constructive comment, the corresponding author in the manuscript is to be considered while ignoring the submission author.

**Introduction:** The introduction effectively justifies the chosen topic and the methods with convincing arguments supported by relevant references, as required.

**Comment:** Line 41: Insulin (please maintain the Case)

**Response:** Thank you for your comment, the suggested changes have been made to the manuscript.

**Comment:** Page line 62-63: Type 1 DM increases Type 2 DM prevalence? May be rewritten as “There is an increasing trend in Type 1 DM worldwide as well”.

**Response:** Thank you for your comment, the suggested changes have been made to the manuscript.

**Comment:** Line 66-67: Repeat info, may be removed

**Response:** Thank you for your comment, the suggested changes have been made to the manuscript.

**Comment:** Line 70-71 Ref 16 – IDF Diabetes Atlas 10th edition may be a better reference for this information

**Response:** Thank you for your comment, the suggested changes have been made in the manuscript to IDF Diabetes Atlas 10^th^ Edition in Reference 16.

**Comment:** Line 79 - Instead of “T2DM” uncontrolled T2 DM may be used

**Response:** Thank you for your comment, the suggested changes have been made to the manuscript as per the below comment to make it concise using scientific terms.

**Comment:** Line 81-82: mention as “Disease” rather than “Damage”

**Response:** Thank you for your comment, the suggested changes have been made to the manuscript.

**Comment:** Line 78-85: May be made briefer and to the point using scientific terms

**Response:** Thank you for your comment, the suggested changes have been made to the manuscript.

**Comment:** Line 94-98 may be moved down after discussion on preferred dual therapy.

**Response:** Thank you for the comment, the section is moved to page no 8, discussion section after the preferred dual therapy as suggested.

**Comment:** Justification may be given for choosing patients on Sitagliptin & Metformin combination, is that the preferred dual therapy in the geographical area?

**Response:** Thank you for your comment, Sitagliptin & Metformin combination is the preferred dual therapy is the preferred choice by endocrinologists in our geographic location.

**Comment:** Inclusion/Exclusion Criteria: It will be helpful to know if patients who are exposed to multiple drug combination before, are also excluded.

**Response:** Thank you for your comment, the suggested changes have been made to the relevant section i.e. inclusion/exclusion criteria in the manuscript.

**Study Procedure:**

**Comment:** Line 138: Term “renal functioning test” may be removed

**Response:** Thank you for your comment, the suggested text is removed from the manuscript.

**Comment:** Line 141: It will be good to know how the third drug is chosen between the three choices.

**Response:** Thank you for your comment, Empagliflozin and Gliclazide were added to the other two oral anti-diabetic medications because both offer cardiovascular benefits. Empagliflozin also has kidney-protective effects, which is important since many patients in Group 1 had high blood pressure and heart disease. Similarly, Glimepiride was chosen for its ability to slow the progression of diabetes and reduce the need for insulin, while also being cost-effective. The choice of a third medication to add to the existing two was based on the patient's overall health, including comorbid conditions, and sometimes the endocrinologist's preference for a specific medication.

**Comment:** Line 147: Grp 2: Please mention the type of Insulin added - analogs, rapid or long acting or mix, etc.

**Response:** Thank you for your comment, the Insulin used in our study was pre-mixed Insulin (70/30), and the same has been added in the method section under the section Group 2 heading.

**Results:**
**Comment:** Table1: The information regarding drug regimen is missing. This information may help to explain the weight gain and glycemic control results. Suggesting to add.

**Response:** Thank you for highlighting this; to avoid confusion, a footnote is added at the end of table 1 showing Group 1 and Group 2 along with details of the treatment regimen.

**Comment:** Data on diabetes age of the patients if available will be significant to interpret the results considering the fact that grp 2 patients are significantly older.

**Response:** Thank you for highlighting this point, the data on how long patients had diabetes was not collected and this could be a possible limitation of this study which is added to the limitation section of the manuscript.

**Comment:** Figure 1: Group1 showed 20% improvement in glycemic control while in Group 2, 30 % of participants showed improvement. The reduction in HbA1c depends on the baseline level. Since number of patients with HbA1c more than 10 are considerable in both the groups, data of ‘more than 8’ may be further grouped and analysed.

**Response:** Thank you for your comment, the patients recruited in the study were having HbA1c > 7% for the last year or more despite using dual OHAs (full doses of Sitagliptin and Metformin). As suggested the data was checked again for further analysis while grouping them in having HbA1c more than 8, the data revealed that only 1 participant/patient in each group was having HbA1c less than 8 i.e. 7.9 in both groups. Due to this further group and analysis will have no impact on the findings. I am hopeful that the honorable reviewer will agree with our point of view.

**Comment:** Line 220-221: Higher the HDL is a positive outcome. Suggested to rewrite the sentence

**Response:** Thank you for your comment, the suggested changes have been made to the manuscript.

**Comment:** Line 240-241: Is this an error? that patients prefer Insulin over OHA? If it is an error, please correct.

**Response:** Thank you for the comment, that was a typo and is removed from the manuscript to avoid confusion to the readers.

**Comment:** The limitations of the study are well-documented and enumerated.

**Response:** Thank you for your comment, we are thankful for sparing time for a thorough review of the manuscript.

Again, thank you for your time and valuable feedback. We believe that the revisions have significantly improved the manuscript and hope that it is now suitable for publication.

Regards

Corresponding author
